# Dissecting the genetic basis underlying drought tolerance at different development stages in soybean

**DOI:** 10.3389/fpls.2026.1874914

**Published:** 2026-08-19

**Authors:** Xiaolei Shi, Rui Tian, Sunlei Ding, Yucheng Wu, Bingbing Lei, Hui Chen, Yangyang Yang, Kai Zeng, Yong Zhan, Qimike Shan, Yongliang Yan

**Affiliations:** 1 Institute of Crop Research/National Central Asian Characteristic Crop Germplasm Resources Medium-term Gene Bank, Academy of Agricultural Sciences of Xinjiang Uyghur Autonomous Region, Urumqi, China; 2 College of Agriculture, Xinjiang Agricultural University, Urumqi, China; 3 Institute of Crops Research, Xinjiang Academy of Agricultural and Reclamation Sciences, Shihezi, China

**Keywords:** causal genes, drought tolerance, genetic Loci, GWAS, soybean

## Abstract

**Introduction:**

Soybean is an indispensable crop supplying protein and oil for humans and animals, and playing an essential role in global food security. Drought represses soybean seed germination, reducing biomass accumulation and even inhibiting yield.

**Methods:**

In order to dissect the genetic components underlying soybean drought tolerance during different development stage, a natural population containing 140 accessions was employed to evaluate seven drought tolerance-related traits under water-welled and drought stress conditions. Subsequently, genome-wide association study (GWAS) was conducted based on 150K single nucleotide polymorphism (SNP) markers of “Zhongdouxin-1”. And the drought tolerance coefficient of seven different traits were analyzed with seven GWAS models.

**Results:**

A total of 1807 significant SNPs were detected across 20 chromosome, including 569 SNPs for germination stage, and 1242 SNPs for seedling stage. Of 569 SNPs identified in germination stage, 354 SNPs on chromosomes 2, 7, 13, 14, and 17 accounting for 62.21%. Among 1242 SNPs found in seedling stage, 869 SNPs on chromosomes 11, 14, 15, 17 and 18 accounting for 69.97%. Moreover, among 1807 significant SNPs, 163 SNPs exhibited pleiotropic effects, of which 23 were located in exon, 21 in intron, 12 in 5’UTR or 3’UTR and 11 in upstream or downstream. Furthermore, 249 stable SNPs were detected by more than four GWAS models. According to these stable SNPs, RNA expression levels and gene annotations, four causal genes (*Glyma.02G080200*, *Glyma.11G056200*, *Glyma.12G188900*, and *Glyma.18G110200*) conferring soybean drought tolerance were detected, which participated in ethylene stimulus response, water deprivation response, and proteolysis.

**Discussion:**

Collectively, 249 stable SNPs, 163 pleiotropic SNPs and four candidate genes identified in present study provided promising molecular resources and reliable foundation for drought resistance improvement and marker-assisted selective breeding in soybean.

## 1 Introduction

Soybean is one of the important legume crop, playing a crucial role in the global food supply chain and food security (Fang et al., 2025). Drought inhibits soybean yield by impairing plant development and delaying key growth processes (Arya et al., 2021). Drought stress arises at different development stages of soybean. During the germination stage, drought stress suppresses seed germination, reduces the germination potential and rate, and ultimately leads to poor seedling emergence in the field (Kong et al., 2025; Du et al., 2025). In the vegetative stage, drought stress not only impairs photosynthesis by causing leaf wilting, scorching, and withering, but also reduces plant height, stem diameter, and biomass accumulation (Falcioni et al., 2025).

Soybean drought tolerance is a complex quantitative trait (Kong et al., 2025; Burner et al., 2026). Multiple studies have identified genetic loci and candidate genes associated with soybean drought tolerance by linkage and association analysis. Sun et al. (2022) identified 22 quantitative trait loci (QTL) for soybean seed germination under 15% PEG-6000 drought stress from a recombinant inbred line (RIL) population (Maple Arrow × Hefeng 25), explaining 7.06 ~ 16.82% of the phenotypic variation explained (PVE). Liu et al. (2020) detected 11 single nucleotide polymorphisms (SNPs) associated with soybean drought tolerance at germination stage via GWAS using a natural population of 259 accessions, which were located on chromosome 5, 6 and 11, with PVE 4.91%~ 7.02%. Aleem et al. (2024) found a significant quantitative trait nucleotide (QTN) (*qGR8-1*) on chromosome 8 linked to soybean relative germination rate under drought stress using a population consisting of 240 soybean accessions. Moreover, Zhao et al. (2022) identified 13 SNPs related to drought tolerance at germination stage, using relative germination potential, relative germination rate and relative germination index in a panel of 410 soybean accessions via GWAS.

In addition to the germination stage, genetic loci associated with drought tolerance at the seedling stage have also been identified. Ouyang et al. (2022) found nine QTLs related to nine soybean drought-related traits, including plant height, main stem node number, branch number and chlorophyll content, by analyzing a RIL population derived from Jindou21 × Zhongdou33, which were located on chromosome 2, 8, 11, 15, 16, and 18. Jiang et al. (2024) detected 33 drought-tolerance QTLs using a RIL population derived from a cross between Lin and Meng, and identified a candidate genes *GmUAA6* located on chromosome 11, which positively regulated the drought tolerance in soybean and *Arabidopsis*. Zhang et al. (2022) identified two stable drought tolerance loci located at 6.21 Mb and 8.86 Mb on chromosome 7, based on evaluations of plant height, root length, and survival rate under drought stress using a RIL population derived from a cross between Zhonghuang 35 and Jindou 21, and a natural population comprising 259 accessions.

Moreover, Burner et al. (2025) identified seven QTLs associated with canopy wilting using a RIL population derived from the fast-wilting variety Benning and the slow-wilting variety PI 603535, and these QTLs were distributed across six chromosomes with PVE 3.2% ~ 10.3%. Kaler et al. (2017) discovered 61 SNPs associated canopy wilting index under drought tolerance via GWAS, and these SNPs were distributed across 19 chromosomes (excluding Chr3). Li et al. (2023) identified 26 SNPs associated with the soybean leaf wilting index through GWAS on a natural population of 188 accessions across three environments (0% PEG-6000, 20% PEG-6000, and natural drought), and these SNPs were located on nine chromosomes, with PVE 12.00% ~ 17.00%. Zhang et al. (2024a; 2024b) identified two significant drought-tolerance associated loci using a panel of 585 soybean accessions through GWAS, which located on chromosomes 8 and 16. Based on these two loci, two causal genes (*GmACO1* and *GmPrx16*) were identified, which acted as positive regulators of drought tolerance in soybean.

Most studies exploring genetic loci for soybean drought tolerance have focused exclusively on one developmental stage. Few studies simultaneously focus on both germination and seedling stages, making it unclear whether the genetic loci governing drought responses overlap or differ between these two developmental phases. Since drought regulatory networks in plants show obvious developmental stage specificity, we hypothesized that most genetic loci conferring drought resistance differ greatly between the soybean germination and seedling stages. To verify this hypothesis, we systematically phenotyped drought-related traits under drought stress at both germination and seedling stages. A total of 140 soybean accessions from a natural population were subjected to phenotyping under both well-watered and drought conditions. At the germination stage, germination rate (GR), germination potential (GP), and germination index (GI) were measured. At the seedling stage, the traits included plant height (PH), stem diameter (SD), shoot fresh weight (SFW) and shoot dry weight (SDW) were measured. The drought tolerance coefficients were evaluated to assess soybean drought tolerance. According to the 150K SNP markers and drought tolerance coefficients of different traits, the genetic components associated with soybean drought tolerance were dissected, laying a theoretical foundation for marker-assisted selection and gene pyramiding in soybean drought tolerance breeding.

## 2 Materials and methods

### 2.1 Plant materials

A natural population of 140 soybean accessions was employed in the present study, which was obtained from the *National Central Asian Characteristic Crop Germplasm Resources Medium-term Gene Bank* (*Urumqi*), consistent with the description in our previous study (Tian et al., 2025). The detailed information was presented in **Supplementary Table 1**.

### 2.2 Assessment of drought tolerance in germination stage

Fifty seeds were selected and sterilized using chlorine gas. Then the seeds were placed in germination boxes (12 cm × 12 cm × 6 cm) lined with two layers of sterilized filter paper. The control group (CK) added 20 mL of sterile distilled water, while the treatment group (drought stress) added 20 mL of 15% PEG-6000 solution (Aleem et al., 2024; Jia et al., 2024). Subsequently, the germination boxes were placed in artificial climate chamber with 25 ± 1 °C and 60% humidity. The number of germination seeds was recorded daily for eight consecutive days, with germination defined as the radicle length exceeding half of the seed length. The experiment was conducted with three replicates. After four and seven days, the GP and GR of soybean accessions were assessed, respectively. The formulas are as follows: GP=Number of germinated seeds on fourth day/total number of seeds sown × 100%, GR=Number of germinated seeds on seventh day/total number of seeds sown × 100%. And GI was calculated by the formula ∑(G_t_/D_t_), where G_t_ represents the number of seeds germination per day, and D_t_ denotes the corresponding day of germination. According to the formula P_d_/P_w_, the drought tolerance coefficient of different traits, including relative germination potential (RGP), relative germination rate (RGR) and relative germination index (RGI) was evaluated, where where P_d_ represents the phenotype value under drought treatment and P_w_ represents the phenotype value under CK conditions.

### 2.3 Evaluation of drought tolerance in seedling stage

The nutritional soil (peat:perlite:leaf mold at a ratio of 5:3:2, v:v:v) was thoroughly mixed with field soil at a 1:1 ratio. Then 2 kg of mixture soil was placed into square plastic pot (18 cm × 18 cm), and 2 L of water was added to each pot. Eight seeds per accession were sown in pot and covered with 2 ~ 4 cm of the mixture soil. Seven days after sowing, seedlings were thinned to three uniform plants per accession. Two treatments were subsequently conducted at the V1 stage, with the control group (CK) maintained at a soil relative water content of ≥65%, and the drought treatment group (drought stress) maintained at 30~40%. After 10 days of drought stress, the drought related traits, including plant height (PH), stem diameter (SD), shoot fresh weight (SFW), and shoot dry weight (SDW), were evaluated for each accession based on “*Specification and Data Standard of Soybean Germplasm Resources Description*”. The experiment was conducted with three replicates. The relative plant height (RPH), relative stem diameter (RSD), relative shoot fresh weight (RSFW), and relative shoot dry weight (RSDW) were also calculated using the formula P_d_/P_w_.

### 2.4 Identification and validation of genetic loci for soybean drought tolerance

The soybean natural population genotyping was performed using the SoySNP 150K array, and SNPs were filtered using the same criteria (missing ratio < 0.15, minor allele frequency ≥ 0.05) as described in our previous study (Tian et al., 2025). The population structure and kinship analysis was estimated via STRUCTURE 2.3.4 and TASSEL 5.0 software, respectively (Tian et al., 2025). To identify the genetic loci responsible for drought tolerance in soybean, GWAS was conducted by the mean value of RGP, RGR, RGI, RPH, RSD, RSFW and RSDW in R package GAPIT3 via seven statistical models (GLM, MLM, MLMM, CMLM, SUPER, BLINK and FarmCPU). The *P* value was set as 0.001. Following the method described in our previous study, the SNPs were attributed to the same locus when their physical distance was less than the linkage disequilibrium (LD) distance (Tian et al., 2025). According to this method, significantly associated SNPs identified in the present study were assigned to different loci. To confirm these loci, the soybean accessions were divided into groups defined by the number of favorable alleles at the the most significant SNP for each locus. Using SPSS version 25.0, regression analysis was implemented to evaluate the association between the cumulative favorable allele count and the corresponding phenotypic data (Huo et al., 2019). The regression analysis between the favorable allele numbers and soybean drought tolerance was performed using GraphPad Prism 9.5 software.

### 2.5 Detecting of the candidate genes for soybean drought tolerance

Firstly, candidate genes were mined within the flanking genomic regions of stable SNPs, which were detected by at least four GWAS models. Then the candidate genes were filtered, based on gene annotation information and biological processes which was obtained from Soybase (https://www.soybase.org/). To detect causal genes responsible for soybean drought tolerance, the gene expressions were analyzed using the published soybean drought tolerance transcriptome data at the seedling stage (GSE98958) and our own transcriptome data at the germination stage. Through DNA mutation, RNA expression level and gene annotations analysis, the causal genes involved in soybean drought tolerance were ultimately identified.

### 2.6 Statistical analysis

The phenotypic variation and analysis of variance (ANOVA) of seven drought tolerance traits were conducted using SPSS version 25.0 software. The phenotypic differences comparisons between different haplotypes were assessed using a two-tailed Student’s *t*-test. Plotting was performed using GraphPad Prism version 9.0 software.

## 3 Results

### 3.1 Phenotypic variation of drought tolerance-related traits

According to the analysis of variance (ANOVA), the significant difference of drought tolerance-related traits were detected among the soybean accessions (**Table 1**). The mean values of RGP, RGR, RGI, RGH, RSD, RSFW and RSDW were 0.67, 0.79, 0.28, 0.54, 0.72, 0.25 and 0.61 respectively (**Table 1**). Further analysis found that the maximum values of RGR, RGI, RGH, RSD, RSFW and RSDW represented a 11.11-fold, 18.00-fold, 2.46-fold, 1.44-fold, 5.40-fold and 2.88-fold increase over their respective minimum values, respectively. The coefficients of variation (*CV*) of RGP, RGR, RGI, RGH, RSD, RSFW and RSDW were 34.84%, 25.34%, 40.89%, 20.63%, 10.90%, 30.51%, 30.23%, suggesting that relatively broad variations existed in soybean natural population (**Table 1**). Moreover, frequency distribution analysis was conducted on seven drought tolerance indices, including RGP, RGR, RGI, RGH, RSD, RSFW and RSDW (**Supplementary Figure 1**). The results showed that all these traits displayed continuous phenotypic variation. The skewness and kurtosis values of all traits were close to zero, supporting an approximately normal distribution. These findings demonstrated that the above traits are quantitative traits and suitable for subsequent genome-wide association analysis (**Table 1**).

**Table 1:** Phenotypic variation analysis of drought-related traits in soybean natural population.

Trait ^a^	Mean	Max	Min	SD	*CV* (%)	Skew.	Kurt.	Sig. ^b^
RGP	0.67	1.00	0.00	0.23	34.84	-0.55	-0.41	**
RGR	0.79	1.00	0.09	0.20	25.34	-1.27	1.00	**
RGI	0.28	0.72	0.04	0.11	40.89	0.44	0.88	**
RPH	0.54	0.86	0.35	0.11	20.63	0.83	0.77	**
RSD	0.72	0.88	0.61	0.08	10.90	0.30	-1.16	**
RSFW	0.25	0.54	0.10	0.08	30.51	0.60	0.35	**
RSDW	0.61	0.95	0.33	0.19	30.23	0.09	-1.34	**

^a^ RGP, relative germination potential; RGR, germination rate; RGI, germination index; RPH, relative plant height; RSD, relative stem diameter; RSFW, relative shoot fresh weight; RSDW, relative shoot dry weight.

^b^ Sig., significance. ** indicates significance difference among soybean accessions at the 0.01 level.

### 3.2 Uncovery of significant loci underlying soybean drought tolerance at germination stage

**Figure 1:**
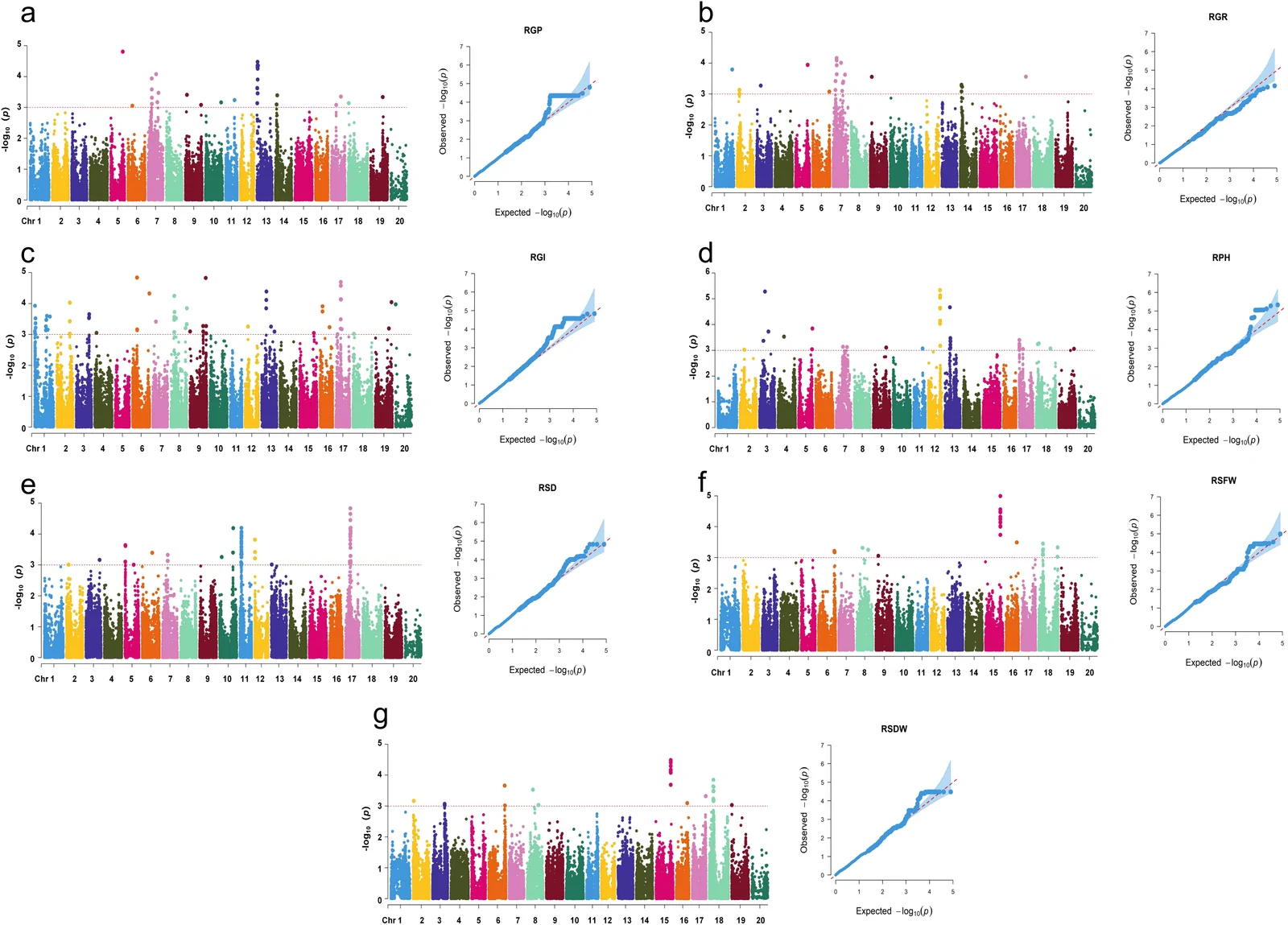
Association analysis of drought tolerance-related traits in soybean natural population. **(a)** Manhattan and QQ plots of relative germination potential. **(b)** Manhattan and QQ plots of relative germination rate. **(c)** Manhattan and QQ plots of relative germination index. **(d)** Manhattan and QQ plots of relative plant height. **(e)** Manhattan and QQ plots of relative stem diameter. **(f)** Manhattan and QQ plots of relative relative shoot fresh weight. **(g)** Manhattan and QQ plots of relative relative shoot dry weight.

To elucidate the genetic basis conferring drought tolerance in soybean during germination stage, the GWAS was used to conduct association analysis on three traits (RGP, RGR and RGI) with multiple models. A total of 569 SNPs were detected, which were located on 20 chromosomes, of which 135 were for RGP, 205 for RGR and 262 for RGI (**Figure 1**, **Supplementary Table 2**). Moreover, 110 SNPs were located on chromosome 13, 69 SNPs on chromosome 7, 60 SNPs on chromosome 17, 59 SNPs on chromosome 2, and 56 SNPs on chromosome 14 accounting for 19.33%, 12.13%, 10.54%, 10.37% and 9.84% of the total SNPs, respectively (**Supplementary Table 2**). Further analysis found that 77 SNPs were detected using more than four models. Among these 77 SNPs, 49 SNPs were mapped to genes. Of which, 13 were located in 5’UTR or 3’UTR, seven in upstream or downstream, which might influence genes expression (**Supplementary Table 2**). Additionally, 15 of the 77 SNPs were located in gene exonic.

### 3.3 Discovery of significant loci conferring soybean drought tolerance at the seedling stage

A total of 1242 significant SNPs associated drought tolerance were detected via multiple GWAS models at the soybean seedling stage, which were located on 20 chromosomes (**Figure 1**, **Supplementary Table 2**). Among them, 441 SNPs were associated with RPH, 456 SNPs associated with RSD, 192 SNPs associated with RSFW, and 286 SNPs associated with RSDW (**Supplementary Table 2**). Moreover, chromosome 18 contained 281 SNPs, chromosome 15 harbored 259, chromosome 11 had 176, chromosome 14 owned 78, and chromosome 17 owned 75, representing 22.62%, 20.85%, 14.17%, 6.28% and 6.04% of the total SNPs, respectively (**Supplementary Table 2**). Further analysis found that 172 SNPs were detected using more than four models. Among these 172 SNPs, 15 was located in the 5’UTR or 3′UTR and 15 was located upstream or downstream. Moreover, 22 SNPs were located in exonic, comprising 16 synonymous mutations, four nonsynonymous mutations, one stopgain mutation and one stoploss mutation. In addition, 97 were intergenic SNPs, 23 were intronic (**Supplementary Table 2**).

### 3.4 Identification of pleiotropic SNPs associated with drought tolerance traits

To facilitate pyramiding breeding in soybean drought tolerance, pleiotropic SNPs were screened from 1807 significant associated SNPs mentioned above. The results show that 163 SNPs exhibit pleiotropic effects, which were distributed across 12 chromosomes. The highest number was found on chromosome 18, with 54 pleiotropic SNPs, followed by chromosomes 11 with 35 pleiotropic SNPs (**Supplementary Table 3**). Among the 163 pleiotropic SNPs, 26 were linked to both RGP and RGR; two had connections with RGI and RGP; two overlapped with RGI, RGP and RGR; 128 displayed connections to RSFW and RSDW; one SNP correlated with RPH and RSD; one showed a relationship with RGI and RSDW; one associated with RGP, RGR and RSDW; two exhibited correlations with RGI, RSFW and RSDW. Further analysis revealed that among these 163 pleiotropic SNPs, 23 were located in exon, 21 in intron, 12 in 5’UTR or 3’UTR, 11 in upstream or downstream, and 96 in intergenic. The identification of these loci provided a theoretical foundation for molecular-assisted breeding facilitating the genetic improvement of drought tolerance in soybean. The discovery of these pleiotropic SNPs facilitated the simultaneous improvement of multiple traits, which in turn enhanced the efficiency of molecular breeding for soybean drought tolerance.

### 3.5 Validation of significant genetic loci for soybean drought tolerance

Based on LD decay distance (150 kb), 249 stable SNPs detected by more than four GWAS model at different stage were divided into 79 loci. To validate the reliability of the significantly associated SNPs identified in this study, 40 homozygous loci located within the significant GWAS signal peaks were used to perform regression analysis between the number of favorable alleles and the phenotypic performance. The results showed that a positive correlation was observed between the number of favorable alleles and the values of RGP, RGR, RGI, RGH, RSD, RSFW and RSDW with r^2^ 0.93, 0.97, 0.77, 0.70, 0.98, 0.87 and 0.92, respectively (**Supplementary Figure 2**). These findings indicate that RGP, RGR, RGI, RGH, RSD, RSFW and RSDW increased with the accumulation of favorable alleles, suggesting soybean accessions harboring more favorable alleles exhibited higher drought tolerance.

### 3.6 Identification candidate genes for soybean drought tolerance

A total of 222 genes were identified within the flanking regions (150 kb) of the stable SNPs detected by more than four GWAS models. (**Supplementary Table 4**). Among these, 37 genes contained exonic SNP mutations, 28 harbored SNP mutations in the 5′UTR or 3′UTR, 20 had upstream or downstream SNP mutations, 145 carried intergenic SNP mutations, and 31 possessed intronic SNP mutations (**Supplementary Table 4**). Further analysis found that 82 genes were identified in the germination stage and 140 genes in the seedling stage. Subsequently, based on soybean drought tolerance transcriptome data from the germination and seedling stages, nine candidate genes for germination-stage tolerance and 21 for seedling-stage tolerance were identified. In addition, four genes (*Glyma.02G080200*, *Glyma.11G056200*, *Glyma.12G188900*, and *Glyma.18G110200*) were selected based on gene annotation information (**Supplementary Table 4**).

**Figure 2:**
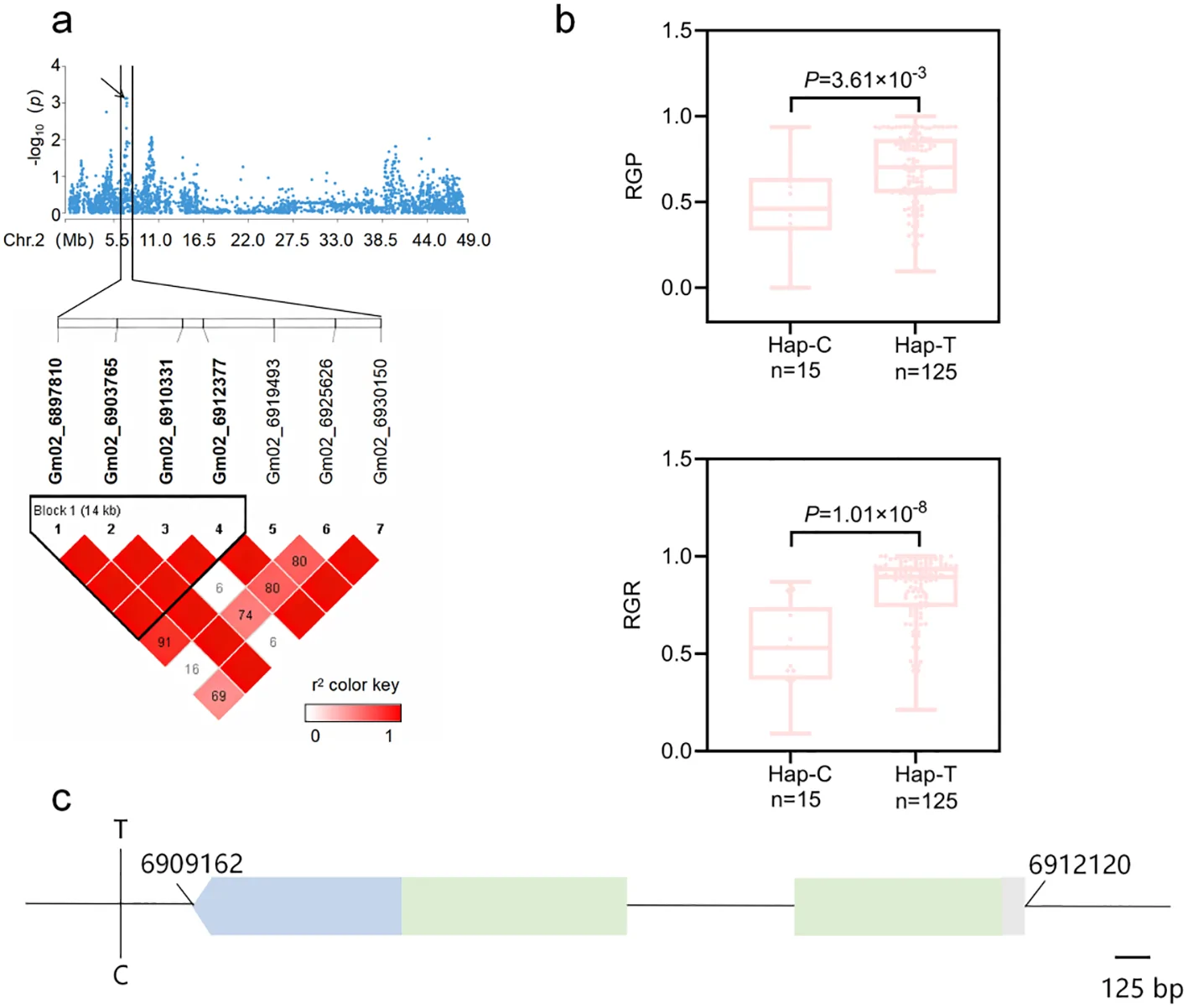
Uncovering of drought tolerance gene *Glyma.02G080200* on chromosome 2. **(a)** GWAS signal on chromosome 2 (top) and LD heatmap (bottom) for *Glyma.02G080200*. Triangular block shows region with strong local LD (r^2^ > 0.94). **(b)** Boxplot for RGP and RGR based on significant associated SNP. In the boxplots, the center line denotes the median, box limits are the upper and lower quartiles; n indicates the accession number with the same genotype. Statistical significance was determined by a two-tailed *t* test. **(c)** Gene structure of *Glyma.02G080200.* Green rectangles indicate exon; gray rectangle and blue pentagon indicate 5’UTR and 3’UTR; vertical black lines indicate SNP mutation.

The candidate gene *Glyma.02G080200* encoded an AP2/ERF transcription factor involved in the ethylene response, and this transcription factor has been reported to respond to drought stress. Gene structure analysis found that *Glyma.02G080200* contained two exons and one intron. Additionally, a significant associated SNP was detected based on the mean value of RGR, which divided the population into two haplotypes (Hap-T and Hap-C), and significant differences in RGR were observed between them (**Figure 2**). Furthermore, the expression level of *Glyma.02G080200* was significantly up-regulated after drought stress during germination and seedling stage, indicating that this gene might be induced by drought stress at soybean different development stages (**Supplementary Table 4**).

**Figure 3:**
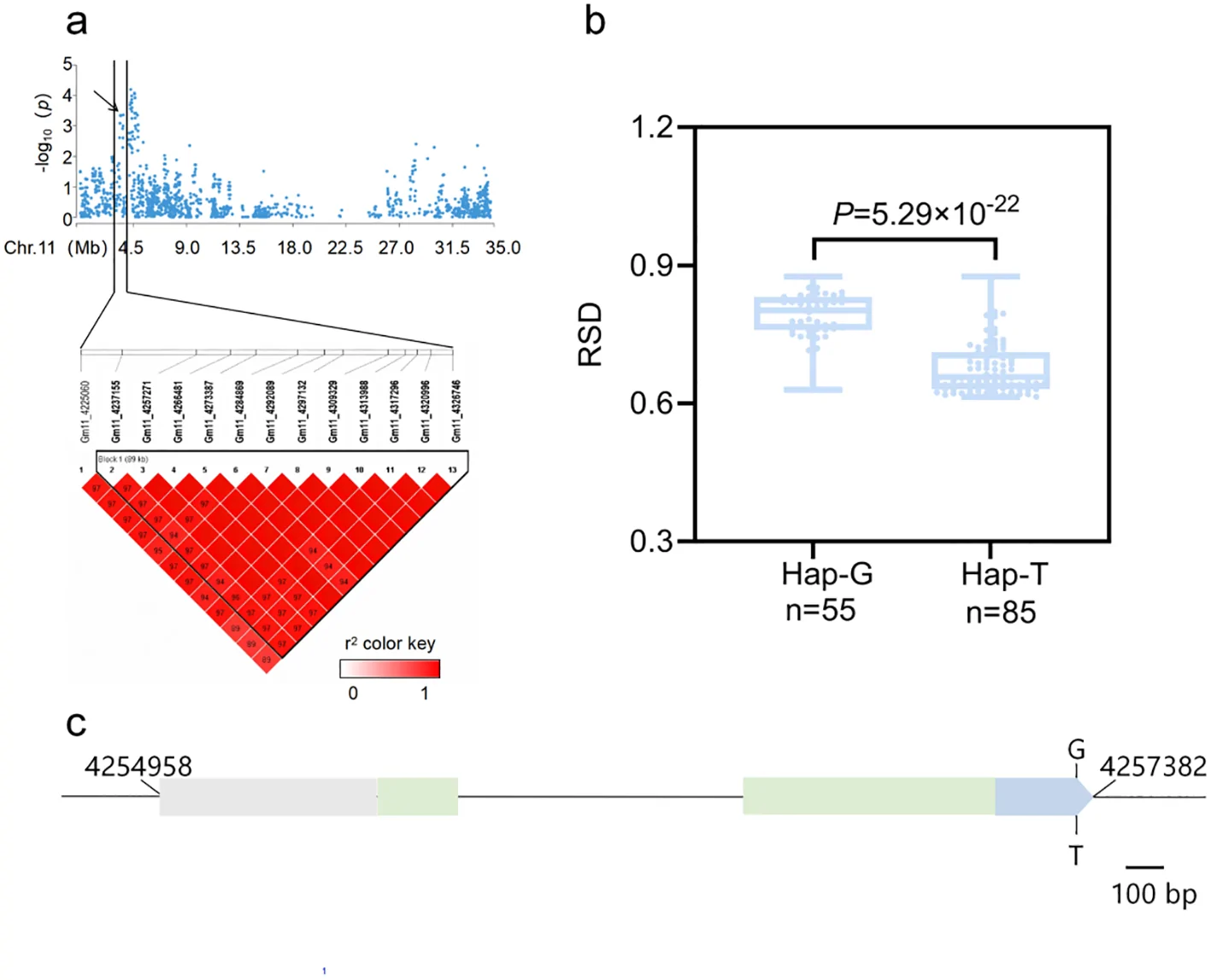
Identification of drought tolerance gene *Glyma.11G056200* on chromosome 11. **(a)** GWAS signal on chromosome 11 (top) and LD heatmap (bottom) for *Glyma.11G056200*. Triangular block shows region with strong local LD (r^2^ > 0.94). **(b)** Boxplot for RSD based on significant associated SNP. In the boxplots, the center line denotes the median, box limits are the upper and lower quartiles; n indicates the accession number with the same genotype. Statistical significance was determined by a two-tailed *t* test. **(c)** Gene structure of *Glyma.11G056200.* Green rectangles indicate exon; gray rectangle and blue pentagon indicate 5’UTR and 3’UTR; vertical black lines indicate SNP mutation.

According to the mean value of RSD, the candidate gene *Glyma.11G056200* was identified. Gene annotation analysis revealed that it encoded heat shock transcription factor 34 and responded to water deprivation (**Supplementary Table 4**). Meanwhile, gene structure analysis revealed that *Glyma.11G056200* contained two exons and one intron, and possessed one SNP in 3′UTR, which divided the natural population into two haplotypes (Hap-G and Hap-T), and a significant difference in RSD was detected between two haplotypes (**Figure 3**). Moreover, the expression level of *Glyma.11G056200* was significantly up-regulated after drought stress during seedling stage, indicating that this gene might be a causal gene responsible for soybean drought tolerance (**Supplementary Table 4**).

In addition, the candidate genes *Glyma.12G188900* on chromosome 12 encoded the U-box domain-containing family protein and mediated response to water deprivation (**Supplementary Table 4**). The expression pattern of *Glyma.12G188900* increased 20-fold after drought stress at seedling stage (**Supplementary Table 4**). A significant difference of RPH was detected between two haplotypes (**Supplementary Figure 3**). Additionally, the candidate gene *Glyma.18G110200* was also identified based on DNA mutations, RNA expression level and gene annotation information, which encoded 60S ribosomal protein L18a and involved in proteolysis. The expression level was increased 30.95-fold after drought stress at seedling stage (**Supplementary Table 4**). A significant difference of RSDW and RSFW were also detected between two haplotypes (**Supplementary Figure 3**).

## 4 Discussion

With global warming intensifying, drought has become increasingly prevalent worldwide, which commonly causes soybean yield losses of 30%~90% (Hussain et al., 2019; Lesk et al., 2016). Soybean is widely recognized as one of the most drought-sensitive crops, with approximately 67 Mha of harvested soybean areas exposed to drought stress between 1983 and 2009 (Dong et al., 2019; Kim et al., 2019). Key soybean developmental stages such as germination and seedling stages are susceptible to drought stress. At germination stage, drought stress impedes seed germination, resulting in reduced and uneven seedling emergence in the field. At the seedling stage, drought brings about decreases in plant height, stem diameter and biomass. Dissecting the genetic basis of drought resistance in soybean and breeding drought-resistant varieties can mitigate drought-induced yield losses, which are critical for sustaining stable soybean yields. However, these approaches relies on the identification of drought-related genetic loci and candidate genes. Genome-wide association study (GWAS) represents a powerful tool to detect elite drought-resistance loci, offering useful gene resources and molecular markers for marker-assisted breeding.

### 4.1 Significant genetic loci conferring drought tolerance in soybean

In this study, we performed a genome-wide association study (GWAS) using a panel of 140 soybean accessions previously genotyped by Tian et al. (2025). Based on seven drought-related traits, a total of 1807 significant associated SNPs were detected, distributed across 20 chromosomes. Compared with previous studies, some genetic loci were consistent (**Supplementary Table 2**). For instance, the SNP Gm01:38948188 on chromosome 1 (associated with RGI in our study) also associated with the germination drought tolerance index (GDTI) reported by Zhao et al. (2022). Seven significant associated SNPs, located in the region 36,397,457~38,775,658 on chromosome 3, overlapped with *Drought tolerance 6-1* (Carpentieri-Pipolo et al., 2011). Additionally, a set of eight SNPs (35,084,659~35,316,232 on chromosome 12) overlapped with *Drought index 1-3* (Du et al., 2009). Canopy wilt serves as a measure of drought stress response in crops. We also compared our significant SNPs with reported QTLs for canopy wilt (**Supplementary Table 2**). Eight SNPs from Gm14:3403414 to Gm14:5385716 overlapped with *Canopy wilt 1-2* (Charlson et al., 2009). In addition, Gm06:47496031 and Gm06:48160274 overlapped with *Canopy wilt 3-12*, six significant SNPs from Gm02:38807632 to Gm02:38898518 overlapped with *Canopy wilt 6-3*, and Gm19:45756154 and Gm19:48145335 overlapped with *Canopy wilt 6-2* (Hwang et al., 2015). Collectively, these findings not only validate the SNPs detected in our study but also provide a foundation for molecular breeding of drought-tolerant soybean.

### 4.2 Causal genes responsible for soybean drought tolerance

Based on DNA mutation, RNA expression and gene annotation, seven candidate genes associated with soybean drought were identified. Among these, the candidate gene *Glyma.02G080200* encoded an AP2/ERF transcription factor. AP2/ERF superfamily members were classified into five categories based on AP2 domain similarity, namely AP2, RAV, SOLOIST, DREB and ERF, which were not only involved in the regulation of plant growth and development, but also played a vital role in the response to drought stress (Ma et al., 2024b; Chen et al., 2022). For example, overexpression of *GmDREB1* significantly enhanced the soybean drought resistance, and resulted in a clearly higher yield than that of the wild type (Chen et al., 2022). In *Arabidopsis* thaliana, overexpression of *OsAP21* led to stronger growth than that of wild-type plants under drought stress and also resulted in a higher expression level of the stress-related gene *RD29B* (Jin et al., 2012). In rice, overexpression of *OsERF71* enhanced drought survival at the vegetative stage and increased total weight gain by 23~42% under drought at the reproductive stage (Lee et al., 2016). And multi-omics sequencing data suggested that *OsERF71* diverted more energy to survival-critical mechanisms related to translation, oxidative response, and DNA replication, while further suppressing energy-consuming mechanisms, such as photosynthesis (Ahn et al., 2017). Furthermore, overexpression of *TaRAP2-13L* in *Arabidopsis* enhanced drought tolerance, while silencing *TaRAP2-13L* in wheat reduced drought tolerance by modulating the ABA signaling pathway and reactive oxygen species homeostasis (Shao et al., 2025). In addition, AP2/ERF also regulated wax biosynthesis to improve drought resistance. The expression of apple AP2/ERF transcription factor *MdSHINE2* in *Arabidopsis* exhibited great change in cuticular wax crystal numbers, morphology and wax composition of leaves and stems (Zhang et al., 2019). In the present study, the expression levels of *Glyma.02G080200* were significantly increased by 1.31- to 29.12-fold under drought stress during germination and seedling stages, implying that *Glyma.02G080200* was induced by drought stress and played a positive role in soybean drought tolerance at both the germination and seedling stages.

Heat shock transcription factor (HSFs) were divided into classes A, B and C, playing significant roles in responding to environmental stresses (Iqbal et al., 2022; Li et al., 2025). In *Arabidopsis* thaliana, overexpression lines of *HSFA1b* showed increased water productivity and harvest index under water-welled and water-limiting conditions (Bechtold et al., 2013). Moreover, the loss-of-function mutant *hsfb1* exhibited increased plant growth and reduced drought stress tolerance compared with the wild-type, while overexpression of *HSFB1* suppressed plant growth and enhanced drought stress tolerance in *Arabidopsis* (Zheng et al., 2025). In alfalfa, the expression of *MsHSF6*, *MsHSF27* and *MsHSF33* gradually increased as the duration of drought, salt stress (Liu et al., 2023). Overexpression of the wheat gene *TaHsfC3–4* in the *Arabidopsis* thaliana quadruple mutant pyr1pyl1pyl2pyl4 complemented its ABA hypersensitive phenotypes and enhanced drought tolerance, indicating that *TaHsfC3–4* plays positive roles in both ABA signaling pathway and drought tolerance (Ma et al., 2024a). In apple, *MdHSFA8a* promoted plant survival under drought conditions and reactive oxygen species scavenging, while it also participating in abscisic acid-induced stomatal closure and promoting the expression of ABA signaling-related genes (Wang et al., 2020). Ectopic overexpression of *ZmHsf05* in rice exhibited better phenotypic performance, higher survival rate, higher proline content, and lower leaf water loss rate, compared with wild-type (Si et al., 2021). However, some HSFs were played a negative roles in response to drought stresses in plant. In maize, the overexpression of *ZmHsf08* resulted in enhanced sensitivity to drought stresses, displaying lower survival rates, higher reactive oxygen species (ROS) levels, and increased malondialdehyde (MDA) contents compared with wildtype (WT) plants (Wang et al., 2021). Overexpression of the white clover gene *TrHSFB2a* in *Arabidopsis* significantly promoted leaf senescence and increased relative electrical conductivity (REC) and MDA contents compared to the wild type under drought conditions, suggesting that *TrHSFB2a* acts as a negative regulator under drought stress (Iqbal et al., 2022). In our study, the candidate gene *Glyma.11G056200* encoding heat shock transcription factor 34 was identified to respond to soybean drought tolerance. The expression level of *Glyma.11G056200* was significantly up-regulated under drought stress at seedling stage, suggesting that this gene might be act as a positive regulator of soybean drought tolerance in soybean.

Ubiquitination was involved in many aspects of plant growth and development, including photomorphogenesis, hormone regulation, senescence, pathogen defense, and responses to abiotic stress (Lyzenga and Stone, 2012; Chung et al., 2013; Dubeaux and Vert, 2017; Zou and Lin, 2021; Gao et al., 2022; Koo et al, 2023). The ubiquitination process involved three steps: (1) the activation of ubiquitin by the ubiquitin-activating enzyme E1, (2) the transfer of activated ubiquitin to the ubiquitin-conjugating enzyme E2, and (3) the ligation of ubiquitin to the target protein by the direct transfer of ubiquitin from E2 or from a protein ligase E3 (Wang et al., 2024). The E2 ubiquitin conjugating enzyme genes (UBC) were widely involved in plant drought resistance. The overexpression of mung bean *VrUBC1* in *Arabidopsis* resulted in heightened sensitivity to drought stress during germination, while conferring better shoot and root growth during seedlings stage (Chung et al., 2013). Overexpressing soybean *GmUBC2* in *Arabidopsis* plants exhibited more tolerant to drought stresses compared with the control plants (Zhou et al., 2010). Overexpression of *StUBC13* in potato transgenic plants led to a reduction in MDA levels and an accumulation of superoxide dismutase (SOD), peroxidase (POD), and catalase (CAT) under drought stress, which contributed to improved drought tolerance (Fu et al., 2024).

In addition, plant U-box (PUB) E3 ubiquitin ligases negatively regulate drought stress responses. For example, heterogeneous overexpression of soybean *GmPUB8* in *Arabidopsis* resulted in decreased drought tolerance, increased sensitivity to osmotic stress-induced inhibition of seed germination and seedling growth, and suppressed mannitol-mediated stomatal closure (Wang et al., 2016). Similarly, overexpressing of *PUB22* and *PUB23* renders *Arabidopsis* hypersensitive to drought stress, whereas loss-of-function of *PUB22* and *PUB22* significantly enhanced drought tolerance, and the *pub22* and *pub23* double mutant even exhibited greater tolerance, indicating that *PUB22* and *PUB23* acted as negative regulators in the water stress response (Cho et al., 2008). Mechanism analyses revealed that PUB22 and PUB23 ubiquitinated 19S proteasome regulatory particle (RP) subunit RPN6, leading to its degradation (Cho et al., 2015). Moreover, PUB22 and PUB23 also interacted with the ABA receptor PYL9 and promoted its degradation (Zhao et al., 2017). In addition, nitrate-responsive transcription factor NLP8 binds the promoter of *PUB23* to activate its expression and thereby negatively regulate drought resistance (Ma et al., 2025). In our study, the candidate genes *Glyma.12G188900* encoded the U-box domain-containing family protein, which was homologous to the PUB23 of *Arabidopsis* thaliana. The expression pattern of *Glyma.12G188900* showed differential expression under drought, indicating that *Glyma.12G188900* might be involved in ubiquitinate substrate proteins for proteasomal degradation under drought conditions.

Additionally, the candidate gene *Glyma.18G110200* was also identified based on DNA mutations, RNA expression level and gene annotation information, which encoded 60S ribosomal protein L18a and involved in proteolysis, which was homologous to the aspartic protease genes of *Arabidopsis*. The aspartic protease (ASP) was involved in plant drought resistance. For example, there are differences in the expression of aspartic protease precursor genes between drought-tolerant and drought-susceptible varieties in bean plants (Cruz de Carvalho et al., 2001). In *Arabidopsis*, overexpression of *ASPG1* could confer drought avoidance for plants by increasing ABA sensitivity in guard cells and simultaneously reducing a reduction in transpirational water loss (Yao et al., 2012). Similarly, overexpressing another aspartic protease gene *APA1* showed a phenotype more tolerant to drought compared with WT, while *apa1* insertional lines were more sensitive to drought stress (Ippolito et al., 2020). Drought stress regulated the activity of the aspartic acid protease *PvAP1* in common bean at both the transcriptional and post-transcriptional levels, and this response occurred earlier and stronger in the drought-susceptible cultivar (Contour-Ansel et al., 2010).

In summary, we identified 1807 SNPs linked to drought tolerance in soybean across the germination and seedling stages using multiple GWAS models. Among these 1807 SNPs, 249 were detected more than four models. These 249 robust SNPs explained the complex genetic regulation of soybean drought resistance and supply valuable molecular marker resources for drought-resistant soybean improvement. Based on these robust SNPs, we identified four candidate genes (*Glyma.02G080200*, *Glyma.11G056200*, *Glyma.12G188900*, and *Glyma.18G110200*) responsible for soybean drought tolerance. The present study supplements novel genetic information to dissect soybean drought tolerance and facilitates marker-assisted molecular breeding.

## 5 Conclusion

In summary, a total of 1807 significant SNPs associated soybean drought tolerance were detected across 20 chromosomes via multiple GWAS models, including 569 SNPs detected for germination stage, and 1242 SNPs identified for seedling stage. Among 1807 significant SNPs, 163 SNPs exhibited pleiotropic effects. In addition, of 1807 significant SNPs, 249 SNPs were detected by more than four GWAS models. According to 249 stable SNPs, RNA expression levels and gene annotations, four causal genes conferring soybean drought tolerance were detected, which participated in ethylene stimulus response, water deprivation response, and proteolysis.

## Data Availability

The datasets presented in this study can be found in online repositories. The names of the repository/repositories and accession number(s) can be found in the article/**Supplementary Material**.
