# Supplementary material for: Dissecting the genetic basis underlying drought tolerance at different development stages in soybean
Source: Front Plant Sci. 2026 Aug 19;17:1874914. doi: 10.3389/fpls.2026.1874914 (PMC13533929; doi:10.3389/fpls.2026.1874914)
Supplement: Supplementary file 1 [file DataSheet1.docx]

**Fig. S1** Histogram of frequency distribution for soybean drought tolerance-related traits. (a) Histogram of frequency distribution of relative germination potential. (b) Histogram of frequency distribution of relative germination rate. (c) Histogram of frequency distribution of relative germination index. (d) Histogram of frequency distribution of relative plant height. (e) Histogram of frequency distribution of relative stem diameter. (f) Histogram of frequency distribution of relative relative shoot fresh weight. (g) Histogram of frequency distribution of relative relative shoot dry weight.

**Fig. S2** Regression analysis of drought tolerance-related traits and elite allele numbers.

**Fig. S3** Haplotype analysis of soybean drought tolerance gene *Glyma.12G188900* (Top)and *Glyma.18G110200* (Bottom).
